# Shifts in the Bacterial Community of Supragingival Plaque Associated With Metabolic-Associated Fatty Liver Disease

**DOI:** 10.3389/fcimb.2020.581888

**Published:** 2020-12-15

**Authors:** Fen Zhao, Ting Dong, Ke-Yong Yuan, Ning-Jian Wang, Fang-Zhen Xia, Di Liu, Zhi-Min Wang, Rui Ma, Ying-Li Lu, Zheng-Wei Huang

**Affiliations:** ^1^ Department of Endodontics, Shanghai Ninth People’s Hospital, College of Stomatology, Shanghai Jiao Tong University School of Medicine, Shanghai, China; ^2^ National Clinical Research Center for Oral Diseases, Shanghai, China; ^3^ Shanghai Key Laboratory of Stomatology & Shanghai Research Institute of Stomatology, Shanghai, China; ^4^ Institute and Department of Endocrinology and Metabolism, Shanghai Ninth People’s Hospital, Shanghai Jiao Tong University School of Medicine, Shanghai, China; ^5^ Computational Virology Group, Center for Bacteria and Viruses Resources and Bioinformation, Wuhan Institute of Virology, Chinese Academy of Sciences, Wuhan, China; ^6^ University of Chinese Academy of Sciences, Beijing, China; ^7^ Shanghai-MOST Key Laboratory of Health and Disease Genomics, Chinese National Human Genome Center, Shanghai, China

**Keywords:** metabolic-associated fatty liver disease, 16S rDNA sequencing, microbial community dysbiosis, insulin resistance, obesity, supragingival plaque

## Abstract

Metabolic-associated fatty liver disease (MAFLD), also known as the hepatic manifestation of metabolic disorders, has become one of the most common chronic liver diseases worldwide. The associations between some oral resident microbes and MAFLD have been described. However, changes to the oral microbial community in patients with MAFLD remain unknown. In this study, variations to the supragingival microbiota of MAFLD patients were identified. The microbial genetic profile of supragingival plaque samples from 24 MAFLD patients and 22 healthy participants were analyzed by 16S rDNA sequencing and bioinformatics analysis. Clinical variables, including indicators of insulin resistance, obesity, blood lipids, and hepatocellular damage, were evaluated with laboratory tests and physical examinations. The results showed that the diversity of the supragingival microbiota in MAFLD patients was signiﬁcantly higher than that in healthy individuals. Weighted UniFrac principal coordinates analysis and partial least squares discriminant analysis showed that the samples from the MAFLD and control groups formed separate clusters (Adonis, *P* = 0.0120). There were 27 taxa with differential distributions (linear discriminant analysis, LDA>2.0) between two groups, among which *Actinomyces* spp. and *Prevotella 2* spp. were over-represented in the MAFLD group with highest LDA score, while *Neisseria* spp. and *Bergeyella* spp. were more abundant in the control group. Co-occurrence networks of the top 50 abundant genera in the two groups suggested that the inter-genera relationships were also altered in the supragingival plaque of MAFLD patients. In addition, in genus level, as risk factors for the development of MAFLD, insulin resistance was positively correlated with the abundances of *Granulicatella*, *Veillonella*, *Streptococcus*, and *Scardovia*, while obesity was positively correlated to the abundances of *Streptococcus*, *Oslenella*, *Scardovia*, and *Selenomonas*. Metagenomic predictions based on Phylogenetic Investigation of Communities by Reconstruction of Unobserved States revealed that pathways related to sugar (mainly free sugar) metabolism were enriched in the supragingival plaque of the MAFLD group. In conclusion, as compared to healthy individuals, component and interactional dysbioses were observed in the supragingival microbiota of the MAFLD group.

## Introduction

Metabolic-associated fatty liver disease (MAFLD), formerly known as non-alcoholic fatty liver disease (NAFLD), refers to a wide spectrum of liver diseases characterized by the presence of hepatic steatosis in the absence of secondary causes, which include simple hepatic steatosis and steatohepatitis (Eslam et al., 2020). Progressive hepatic fibrosis is a hallmark of advanced steatohepatitis and can lead to cirrhosis, liver failure, and hepatocellular carcinoma (Sheka et al., 2020). MAFLD is an emerging public health concern worldwide, with a global pooled prevalence, by imaging, of 25.24% (Chalasani et al., 2018). The etiology of MAFLD is described as a complex hepatic manifestation of metabolic disorders, and the “multiple-hit” theory has been widely accepted as a potential mechanism involving insulin resistance (IR), obesity, chronic low grade inflammation, a sedentary lifestyle, regular consumption of a high fat diet, adipose tissue dysfunction, genetic factors, and gut microbial dysbiosis (Tiniakos et al., 2010; Buzzetti et al., 2016). With the increasing acknowledgement of the oral microbiome as a source of systemic inflammation, dysbiosis in the oral microbiome had been closely associated with metabolic diseases, including MAFLD (Acharya et al., 2017). A previous study found that the frequency of *Porphyromonas gingivalis* in the oral cavity is significantly higher in MAFLD patients as compared with health subjects (Yoneda et al., 2012). In another study, intravenous injection of sonicated *P. gingivalis* caused impaired glucose tolerance, IR, and liver steatosis in C57BL/6J mice fed a high-fat diet (Sasaki et al., 2018). However, because relatively few oral resident microbes have been the focus of previous studies, changes to the oral microbial community in patients with MAFLD remain unknown.

As a common site of oral sampling, the microbial profile of supragingival plaque is thought to reflect the health status of the host, such as gestation (Lin et al., 2018), type 2 diabetes (Artese et al., 2015), and other conditions (Espinoza et al., 2018). As compared with subgingival plaque, the supragingival microbiota can be acquired more easily and with less discomfort to the host (Lin et al., 2018). Therefore, sampling of the supragingival microbiota presents a promising method to assess oral and systematic health, especially for a large-scale health census. To the best of our knowledge, the present study is the first to assess the association between supragingival microbiota and MAFLD.

In the present study, supragingival plaques were obtained from 24 patients with MAFLD and 22 healthy controls. Illumina MiSeq PE300 sequencing and bioinformatics analysis were employed to identify changes to the microbial profiles and inter-taxa relationships of the supragingival plaque of MAFLD patients. Specific microbial genera correlated with MAFLD and related clinical indexes were also identified. Furthermore, potential functional alterations to the supragingival microbiome were predicted based on the sequencing results. These findings would provide a deeper understanding of the oral ecological dysbiosis associated with MAFLD.

## Materials and Methods

### Study Population

The study protocol was approved by the Ethics Committee of Shanghai Ninth People’s Hospital affiliated with Shanghai Jiao Tong University, School of Medicine (Shanghai, China) (approval no. SH9H-2019-T295-1) and conducted in accordance with the tenets of the Declaration of Helsinki. Written informed consent was obtained from all participants prior to enrollment.

The study participants were recruited from a health census and assigned to one of two groups: the MAFLD group, consisting of 24 persons diagnosed with MAFLD *via* upper abdomen ultrasonography and other clinical examinations (Eslam et al., 2020), or a control group, consisting of 22 persons with normal findings by upper abdomen ultrasonography. Age and sex in the participants of the two groups were matched. For each participant, upper abdomen ultrasonography was performed successively by two experienced sonographers and those with the same diagnosis were enrolled.

The exclusion criteria were as follows: (i) the presence of other liver diseases (e.g., viral hepatitis, autoimmune hepatitis, and hepatolenticular degeneration); (ii) drug-induced hepatic steatosis (e.g., tamoxifen, amiodarone, valproate, methotrexate, and glucocorticoids); (iii) other factors that may cause hepatic steatosis (e.g., long-term total parenteral nutrition, inflammatory bowel disease, celiac disease, hypothyroidism, Cushing’s syndrome, lipoprotein deficiency, lipid-atrophic diabetes, etc.); (iv) use of lipid-lowering drugs within the past 6 months; (v) type 1 or type 2 diabetes; (vi) current oral disease (e.g., untreated oral abscess, precancerous lesions, oral cancer, oral fungal infection, missing more than eight teeth, etc.); and (vii) other conditions (e.g., pregnant or lactating women, long-term heavy smoking, use of antibiotics for more than 5 days within the past 6 months, severe acute episode of a systematic disease, abnormal thyroid function, familial hyperlipidermia, etc.).

### Acquisition of Clinical Variables

All participant demographics were retrieved from self-reported questionnaires and fasting blood samples were collected to detect clinical levels of total cholesterol (TC), total triglycerides (TG), low-density lipoprotein cholesterol (LDL-C), high-density lipoprotein cholesterol (HDL-C), alanine aminotransferase (ALT), aspartate aminotransferase (AST), gamma glutamyl transpeptidase (GGT), fasting plasma glucose (FPG), and fasting serum insulin (FSI). As an approximation of IR, the Homeostatic Model Assessment for Insulin Resistance (HOMA-IR) equation was calculated as HOMA-IR = FPG (mmol/L) × FSI (mU/L)/22.5 (Matthews et al., 1985). The waist circumference, weight, height, and body mass index (BMI) of each participant were acquired *via* physical examination. The unpaired Student’s t-test was applied for analysis of all clinical variables with an exception of “sex,” which was analyzed using the Yates’ continuity corrected chi-squared test.

### Dental Examination and Supragingival Plaque Collection

An abbreviated dental exam was performed on all the participants by the same dentist using a periodontal probe. At least 9 teeth present and >30% of the probed sites with attachment loss ≥1 mm were diagnosed as periodontitis (Armitage, 1999). The prevalence of periodontitis between the two groups was analyzed using the Yates’ continuity corrected chi-squared test. Supragingival plaque was collected before eating in the morning in accordance with the methods described in the Manual of Procedures for the Human Microbiome Project (https://www.hmpdacc.org/hmp/doc/HMP_MOP_ Version12_0_072910.pdf) with minor modifications. The index teeth (#3, #9, #12, #19, #25, and #28) were isolated with cotton rolls and dried under a gentle stream of air. A sterile sickle scaler was used to collect the supragingival plaque from the buccal surfaces of the index teeth. Then, the scaler tips were immersed in 300 µl of sterile normal saline contained in a sterile Eppendorf tube for 5–10 s with slight shaking and the surface of the scaler was wiped off on the inside edge of the tube. When the supragingival plaques of all index teeth were obtained, the Eppendorf tubes were sealed, marked, and kept frozen in liquid nitrogen until DNA extraction.

### DNA Extraction, Amplification, and High-Throughput Sequencing

Total bacterial genomic DNA was extracted from the collected supragingival samples using the QIAamp DNA Mini Kit (Qiagen, Valencia, CA, USA) in accordance with manufacturer’s protocols. The concentration and purification of the extracted DNA were determined using a NanoDrop 2000 UV-vis spectrophotometer (Thermo Scientific, Wilmington, DE, USA), while DNA quality was checked by 1% agarose gel electrophoresis.

The 16S rDNA hypervariable V3–V4 region was PCR-amplified with the forward primer 338F (5′-ACTCCTACGGGAGGCAGCAG-3′) and the reverse primer 806R (5′-GGACTACHVGGGTWTCT AAT-3′) using a GeneAmp™ PCR System 9700 (Applied Biosystems, Carlsbad, CA, USA). The parameters of the PCR reactions have been described in a previous study (Tong et al., 2019). Each PCR reaction was performed in triplicate and all resulting PCR products were extracted from 2% agarose gels and then further purified using the AxyPrep DNA Gel Extraction Kit (Axygen Biosciences, Union City, CA, USA) and quantified using a QuantiFluor^®^ Single-Tube Fluorometer (Promega Corporation, Madison, WI, USA) in accordance with the manufacturer’s instructions. Purified amplicons from different samples were pooled in equimolar concentrations and paired-end sequenced on an Illumina MiSeq PE300 sequencing platform (Illumina, Inc., San Diego, CA, USA).

### Data Processing and Bioinformatics Analysis

Raw fastq files were quality-filtered using the Trimmomatic read trimming tool (https://kbase.us/) and merged using FLASH software (version 1.2.11; https://ccb.jhu.edu/software/FLASH/index.shtml) in accordance with the criteria described in a previous report (Tong et al., 2019). After trimming, operational taxonomic units (OTUs) were clustered at a similarity cutoff value of 97% using the UPARSE algorithm (version 7.1; http://drive5.com/uparse/). The taxonomy of each 16S rRNA gene sequence was analyzed with RDP Classifier algorithm (http://rdp.cme.msu.edu/) against the Silva 16S rRNA database (Release132 http://www.arb-silva.de), the confidence threshold was set to 70%.

Alpha diversity indexes were calculated using MOTHUR software for describing and comparing microbial communities (version 1.30.2; https://www.mothur.org/wiki/Download_mothur) and rarefaction curves were constructed at an inter-sequence similarity value of 97% using the QIIME bioinformatics pipeline (version 1.9.1; http://qiime.org/install/index.html). Bar plots were generated to visualize the species composition of all samples at the phylum and genus levels, and heat maps at the genus level were constructed using the R platform (version 3.6.1). Weighted UniFrac principal coordinates analysis (PCoA), nonparametric multivariate analysis of variance (Adonis), and partial least squares discriminant analysis (PLS-DA) were performed to identify differences in species composition between the MAFLD and control groups using QIIME. The linear discriminant analysis (LDA) effect size (LEfSe; http://huttenhower.sph.harvard.edu/galaxy) was applied to identify the most discriminatory taxa between the groups at the phylum to genus levels. Taxa with logarithmic LDA scores of >2.0 were regarded as discriminative species. Co-occurrence networks of the 50 most abundant genera of each group were demonstrated using NetworkX (version 1.9.1). Spearman’s correlation coefficients were calculated and those with a ∣ρ∣ value of >0.5 and a probability (*P*) value of < 0.05 were visualized. Spearman’s correlation coefficients among the clinical variables and the top 50 abundant genera of the supragingival microbiome were calculated, and the results were visualized as heat maps *via* the R platform. Furthermore, the bioinformatics software package Phylogenetic Investigation of Communities by Reconstruction of Unobserved States (PICRUSt2, version 1.1.0; http://picrust.github.io/picrust/) was used to predict the functional pathways of each group according to the Kyoto Encyclopedia of Genes and Genomes (KEGG). Statistical differences in top 30 abundant KEGG level 2 pathways and top 50 abundant KEGG level 3 pathways between two groups were determined by Wilcoxon rank-sum test with a Benjamini-Hochberg false discovery rate (FDR) correction to adjust P values for multiple testing.

## Results

### Subject Characteristics

The demographic and clinical characteristics of the participants are summarized in **Table 1**. There were no significant differences in age, sex, blood pressure, heart rate and prevalence of periodontitis between the two groups. Subjects in the MAFLD group had relatively higher TG levels and lower HDL-C levels, and thus had higher TG/HDL-C ratios than the control group. Moreover, ALT and GGT levels were relatively higher in the MAFLD group than the control group. Because subjects with diabetes were excluded, there was no significant difference in FPG levels between the two groups, but FSI and HOMA-IR were significantly increased in the MAFLD group. Waist circumference was significantly higher in the MAFLD group, illustrating visceral adiposity was ubiquitous in MAFLD patients.

**Table 1:** Demographic and clinical characteristics of the study participants.

Parameter	Health(n=22)	MAFLD(n=24)	*P*
Age (years)	52.91 ± 4.25	52.75 ± 4.39	0.9013
Sex (M: F)	11: 11	13: 11	0.9898
TC (mmol/L)	5.77± 0.78	5.47 ± 0.88	0.2254
TG (mmol/L)	1.55 ± 1.04	2.51 ± 1.28	0.0083*
LDL-C (mmol/L)	3.41± 0.52	3.28± 0.66	0.4735
HDL-C (mmol/L)	1.40 ± 0.36	1.10± 0.21	0.0011*
ALT (U/L)	22.68 ± 12.99	40.25 ± 27.55	0.0092*
AST (U/L)	31.5 ± 18.13	38.21 ± 19.09	0.2292
AST/ALT	1.52 ± 0.66	1.11 ± 0.43	0.0142*
GGT (U/L)	24.50 ± 21.16	57.42 ± 52.11	0.0084*
FPG (mmol/L)	5.01 ± 0.46	5.18+0.56	0.3196
FSI(mU/L)	4.02 ± 2.68	6.84 ± 2.68	0.0015*
HOMA-IR	0.94 ± 0.59	1.58 ± 0.66	0.0013*
Weight (kg)	62.20 ± 11.75	68.23 ± 9.64	0.0631
BMI	24.13 ± 3.00	25.79 ± 2.61	0.0502
WC(cm)	81.84 ± 9.45	89.79 ± 6.88	0.0020*
SBP (mm Hg)	139.00 ± 26.88	136.46 ± 19.65	0.7144
DBP (mm Hg)	82.77 ± 14.93	82.79 ± 12.17	0.9962
HR Periodontitis (%)	76.09 ± 8.38 81.82	73.71 ± 10.25 79.17	0.3954 0.8843

Data are presented as the mean ± standard deviation. *P < 0.05. All clinical and demographic data were analyzed using the unpaired t-test with an exception of “sex” and “periodontitis”, which were analyzed with the Yates’ continuity corrected χ2 test. Abbreviations: ALT, alanine aminotransferase; AST, aspartate aminotransferase; BMI, body mass index; DBP, diastolic blood pressure; FPG, fasting plasma glucose; FSI, fasting serum insulin; GGT, gamma glutamyl trans-peptidase; HDL-C, high-density lipoprotein cholesterol; HOMA-IR, homeostatic model assessment of insulin resistance [HOMA-IR = FPG (mmol/L) × FSI (mU/L)/22.5]; HR, heart rate; LDL-C, low-density lipoprotein cholesterol; SBP, systolic blood pressure; TC, total cholesterol; TG, total triglycerides; WC, waist circumference.

### Bacterial Diversity and Community Structure of Supragingival Microbiota

The concentration and purification of the extracted DNA met the requirements for further experiments (**Table S1**), results of 2% agarose gel electrophoresis revealed that the PCR products of the extracted DNA was qualified to further sequencing and analysis (**Figure S1**). A total of 2,318,404 high quality sequences were produced, with an average of 50,507 ± 11,212 sequences per sample. In total, 23 phyla, 37 classes, 92 orders, 163 families, 339 genera, 628 species, and 1,021 OTUs were identified after taxonomic assignment of the sequences. All 16S rRNA gene sequences were submitted to the NCBI Sequence Read Archive (SRA) under bioproject accession PRJNA645880 (http://www.ncbi.nlm.nih.gov/sra).

As shown in **Table 2**, the MAFLD group had a higher Shannon index and lower Simpson index, suggesting the diversity of supragingival microbiota was higher in the MAFLD group than the control group (**Table 2**). Good’s coverage indexes, which were close to 1 (**Table 2**), and rarefaction curves based on OTU levels (**Figure S2**) reached saturation plateaus, indicating that the sequencing depths were sufficient to represent the majority of the microbiota in both groups.

**Table 2:** α-diversity of supragingival microbiota in the MAFLD and Control groups.

	MAFLD	Health	*P*
Ace	318.96 ± 76.36	285.74 ± 82.16	0.1561
Chao 1	324.99 ± 81.69	287.67 ± 77.17	0.0969
Shannon	4.02 ± 0.27	3.80 ± 0.39	0.0303*
Simpson	0.04 ± 0.01	0.05 ± 0.02	0.0192*
Coverage	0.9983 ± 0.0006	0.9987 ± 0.0006	0.1319

Results are presented as the mean ± standard deviation. *P < 0.05. All α-diversity estimators were analyzed using the Wilcoxon rank-sum test.

**Figure 1:**
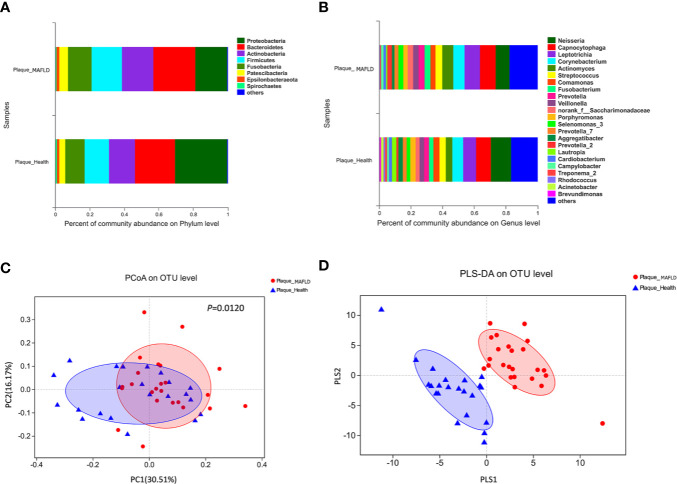
Comparison of supragingival microbiota structures in the metabolic-associated fatty liver disease (MAFLD) and control groups. **(A)** Community structures at the phylum level. **(B)** Community structures at the genus level. **(C)** Weighted UniFrac principal coordinates analysis (PCoA) at the operational taxonomic unit (OTU) level. **(D)** Partial least squares discriminant analysis (PLS-DA) at the OTU level.

The taxa abundance of the supragingival microbiome from the two groups at the phylum and genus levels are depicted in **Figures 1A, B**, respectively. In general, the dominant taxa of the two communities were similar and consistent with the core species of the supragingival microbiome. The core phyla of all samples from both groups consisted of *Bacteroidetes, Proteobacteria, Actinobacteria, Firmicutes, Fusobacteria, Patescibacteria, Epsilonbacteraeota*, and *Spirochaetes*. Of the top 10 abundant genera, the MAFLD group had higher proportions of *Capnocytophaga, Leptotrichia, Corynebacterium, Actinomyces, Streptococcus, Fusobacterium, Prevotella*, and *Veillonella*; while *Neisseria* and *Comamonas* were more prevalent in the control group. The abundances of the top 50 abundant genera in each sample are displayed in the heat map presented in **Figure S3**.

Weighted UniFrac PCoA at the OTU level was employed to evaluate the similarity of the bacterial communities between the two groups. The results indicated that although samples from the two groups partly overlapped, there was a tendency of separation along the PC1 axis (**Figure 1C**). The results of Adonis (*P* = 0.0120) based on weighted UniFrac distances further verified the existence of significant differences in the overall structures of the supragingival microbiota of the two groups. PLS-DA, a supervised analysis suitable for high dimensional data, showed separate clustering of the samples from the MAFLD and control groups (**Figure 1D**), further demonstrating remarkable differences in the supragingival microbiota between the two groups.

### Alterations of the Supragingival Microbial Phylotypes/Inter-Genera Relationship Associated With MAFLD

**Figure 2:**
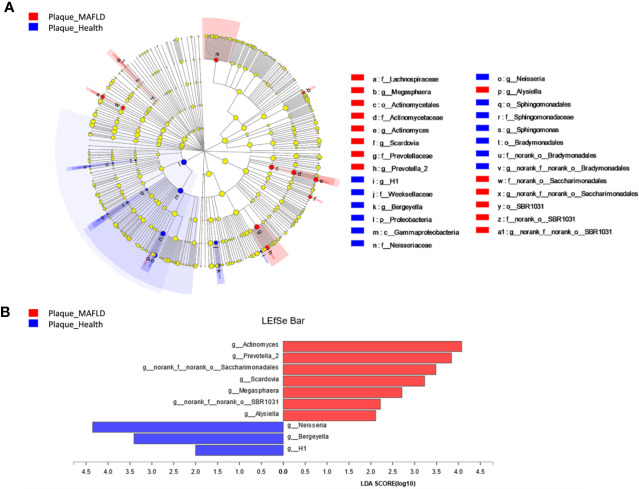
Bacterial phylotypes with altered abundances associated with MAFLD. **(A)** A cladogram of taxonomic representation based on LEfSe. Red indicates enrichment in samples from MAFLD patients and blue indicates the taxa enriched in samples from healthy controls. **(B)** A histogram of the logarithmic linear discriminant analysis (LDA) scores were calculated for the selected genera (logarithmic LDA>2.0, *P*<0.05).

**Figure 3:**
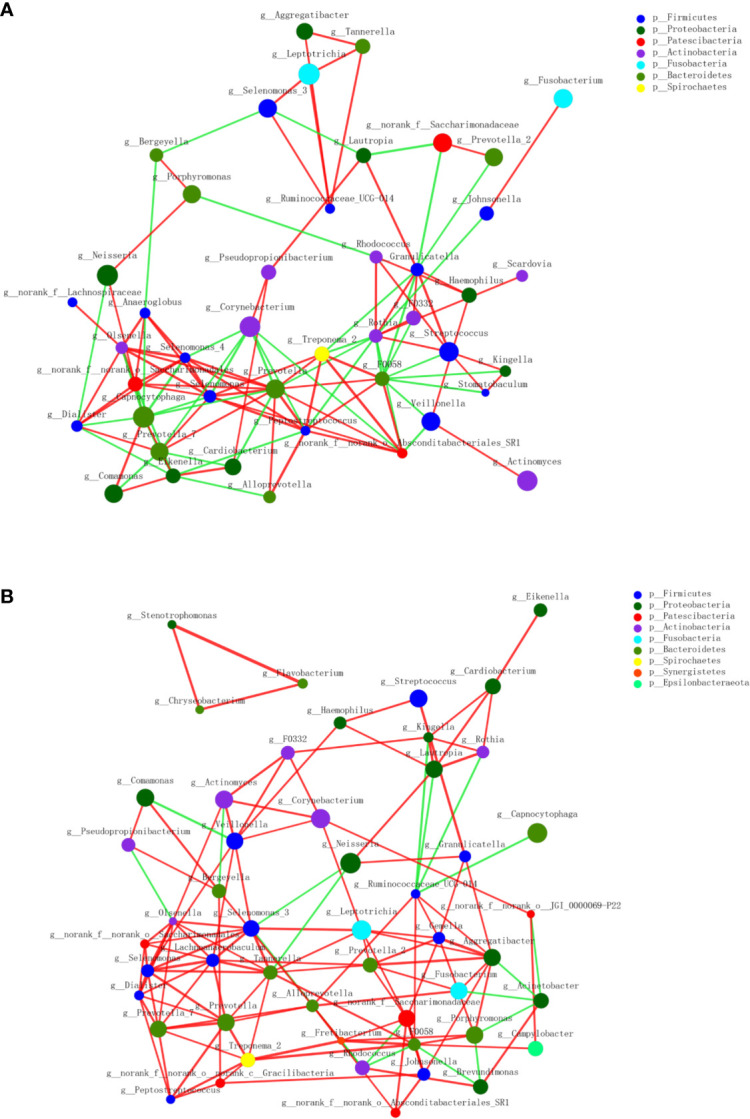
Co-occurrence networks of the top 50 abundant genera in supragingival microbiota. The MAFLD group is shown in **(A)** and Health group in **(B)**. The size of the node indicates the mean relative abundance of the corresponding genus. The same color represents genera belonging to the same phylum. The thickness of the connecting lines corresponds to the coefﬁcient values (*P* < 0.05). The red and green lines indicate positive and negative correlations, respectively.

A circular cladogram based on the LEfSe results demonstrated differentially abundant taxa between the two groups (**Figure 2A**). Genera with logarithmic LDA scores of >2.0 (*P* < 0.05) are plotted in **Figure 2B** and the selected taxa in other taxonomic level (from phylum to family) were shown in **Figure S4**. Briefly, there were 27 taxa with differential distributions between the two groups. At the genus level, *Actinomyces, Prevotella 2, Scardovia, Megasphaera*, and *Alysiella* were more abundant in the MAFLD group, while *Neisseria, Bergeyella, Sphingomonas*, and *H1* were over-represented in the control group. Co-occurrence networks of the top 50 abundant genera of the two groups were depicted in **Figures 3A, B**. In general, the taxa within the main bacterial cluster (>5 nodes and connected with intense lines) had stronger and more complex interrelationships in the MAFLD group.

### Correlations Between Clinical Variables and Supragingival Microbiota

**Figure 4:**
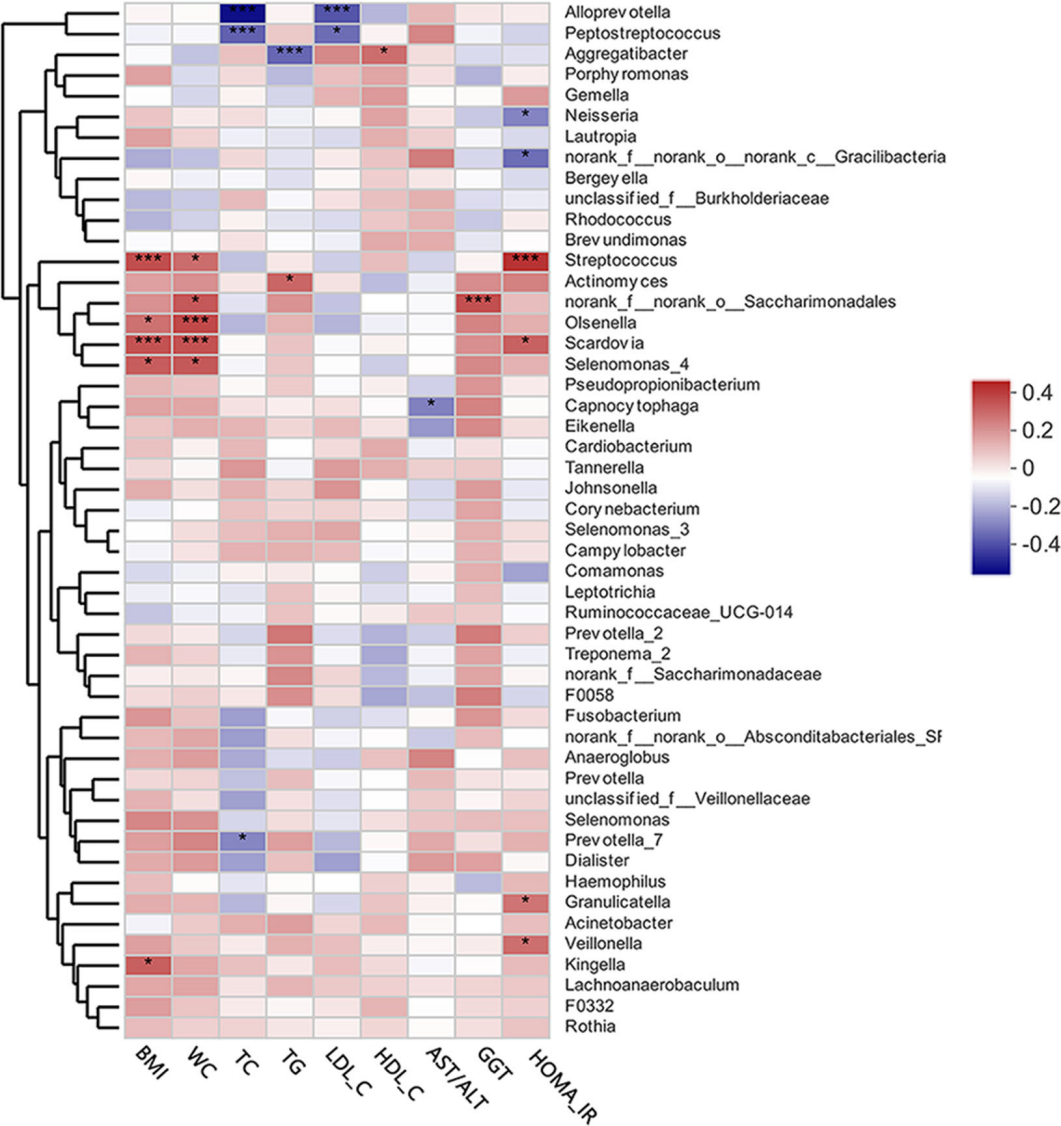
A heat map of Spearman’s correlation analysis of the top 50 abundant supragingival microbiota and clinical variables. The right side of the legend shows the color range of different R values. Species clustering trees are presented on the left side of the heat map. *P < 0.05; ***P < 0.001.

The heat map presented in **Figure 4** depicts the correlations between the 50 most abundant bacterial genera and single clinical variables based on the Spearman’s correlation coefficients. Of the top 50 abundant genera, HOMA-IR showed positive correlations with S*treptococcus, Scardovia, Granulicatella*, and *Veillonella*, but a negative correlation with *Neisseria*. *Alloprevotella*, and *Peptostreptococcus* were extremely significantly negatively correlated with TC and LDL-C levels. *Aggregatibacter* was negatively correlated with TG levels, but positively correlated with HDL-C levels. *Streptococcus, Oslenella, Scardovia*, and *Selenomonas* were significantly positively correlated with BMI and waist circumference. AST/ALT and GGT, two indicators of hepatocellular damage, were negatively correlated with *Capnocytophaga*.

### Predictive Metagenome Functional Profiling of the Supragingival Microbiomes of the MAFLD and Control Groups

**Figure 5:**
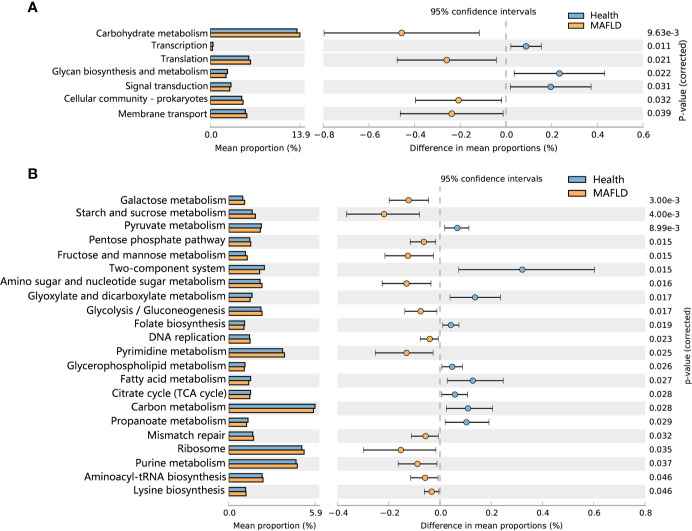
Predictive metagenome functional profiling of supragingival microbiome of the MAFLD and control groups. **(A)** Relative abundance of predictive metagenome functional profiling of the top 20 abundant KEGG level 2 pathways (pathways with *P*_(FDR)_<0.05 are shown). **(B)** Relative abundance of predictive metagenome functional profiling of the top 50 abundant KEGG level 3 pathways (pathways with *P*_(FDR)_<0.05 are shown).

To detect functional differences in the supragingival microbiomes of the MAFLD and control groups, PICRUSt 2 was employed to predict the metagenome functional contents based on the 16S rRNA datasets. Statistically significant differences (*P* < 0.05) in KEGG pathways were calculated with the Wilcoxon rank-sum test with FDR correction. As shown in **Figure 5A**, among the top 30 abundant KEGG level 2 pathways, Carbohydrate metabolism, Translation, Cellular community–prokaryotes and Membrane transport were significantly increased in the MAFLD group. At KEGG pathway level 3, starch and sucrose metabolism, fructose and mannose metabolism, and galactose metabolism were enriched in the MAFLD group. Meanwhile, a total of 9 pathways among the top 50 abundant KEGG level 3 pathways, including carbon metabolism, pyruvate metabolism, and the citrate cycle, were significantly decreased in the MAFLD group (**Figure 5B**).

## Discussion

The oral microbiota is attracting increased attention because of probable associations with metabolic disorders (Si et al., 2017; Chen et al., 2020; Wei et al., 2020). The associations between the oral microbiota and metabolic disorders can be explained in the following two aspects. On the one hand, microbial dysbiosis in the oral cavity is a source of systemic inflammation, which could lead to chronic low-grade inflammation and adversely affects the metabolic health of the host (Acharya et al., 2017). On the other hand, the oral microbiota can influence the composition of the gut microbiome, which plays important roles in metabolic health (Segata et al., 2012; Tremaroli and Bäckhed, 2012; Arimatsu et al., 2014; Li et al., 2019). Due to the anatomical position, about 10^11^ bacteria are swallowed from the oral cavity to the stomach every day (Segata et al., 2012), cultivation and sequencing techniques have also substantiated the association between the oral and gut microbiomes: Arimatsu et al. reported that oral administration of *P. gingivalis* significantly altered the *Firmicutes/Bacteroidetes* ratio, a significant index to evaluate the health status of the gut microbiome (Arimatsu et al., 2014); Li et al. found that the oral microbiota could overcome physical barriers and colonize the gut in gnotobiotic mice (Li et al., 2019). These findings acknowledged that the oral microbiota plays an important role in the development of metabolic diseases *via* “oral-gut axis”. For MAFLD, although some oral resident microbes have been associated with the development of it (Yoneda et al., 2012; Sasaki et al., 2018), there has been no microbiome-wide association study of the association between the development of MAFLD and oral microbial ecology. Shaped by the health status of the host, supragingival plaque has been related to various metabolic disorders. For example, Hintao et al. reported significant differences in the microbial profiles of supragingival plaque between subjects with and without diabetes (Hintao et al., 2007); La Monte et al. found that metabolic syndrome was significantly associated with supragingival plaque (odds ratio = 1.74; 95% confidence interval = 1.22–2.50) (LaMonte et al., 2014). Considering supragingival plaque can be obtained with minimal discomfort and risk (Lin et al., 2018), it was collected in this study to explore the ecological shifts of oral microbiota in MAFLD patients. By screening with strict inclusion/exclusion criteria and matching of confounding factors, the differences among the participants were minimized as much as possible in order to focus on compositional and structural differences of the supragingival microbiota in MAFLD patients.

The diversity of the supragingival microbiota of each group was determined using alpha diversity estimators. It is generally acknowledged that microbial diversity reflects the health status of the host. For example, decreased diversity of gut microbiota indicates functional or metabolic disorders in the host (Kelly et al., 2016), while increased diversity of oral microbiota is reported to imply poor oral (Camelo-Castillo et al., 2015; Takeshita et al., 2016) and holistic health (Armingohar et al., 2014; Si et al., 2017), because in a state of poor oral health, gingival bleeding provides a richer nutrient source (Segata et al., 2012). In the present study, increased diversity (lower Simpson index and higher Shannon index) of the supragingival microbiota in the MAFLD group was observed, suggesting possible alterations to the nutritional status of supragingival plaque in MAFLD patients. Consistent with previous studies (Utter et al., 2016; Lin et al., 2018), the core phyla identified in the present study included *Proteobacteria*, *Bacteroidetes*, *Firmicutes*, *Actinobacteria*, and *Fusobacteria*, which accounted for 93.83% and 92.37% of the supragingival microbiomes of the control and MAFLD groups (**Figure 1A**), respectively. Similarly, although the proportions differed, the majority of the observed genera (including *Capnocytophaga*, *Leptotrichia*, *Corynebacterium*, *Actinomyces*, *Streptococcus*, *Fusobacterium*, *Prevotella*, *Veillonella*, *Neisseria*, and *Comamonas*) existed in both groups, thereby also supporting the core genera of the supragingival microbiota (Lin et al., 2018). A lower *Firmicutes/Bacteroidetes* ratio is considered as a healthy trait in both the oral cavity and gut (Chen et al., 2020). In the present study, the *Firmicutes/Bacteroidetes* ratio was lower in the supragingival plaque of the control group as compared to the MAFLD group (61.41% vs. 72.38%, calculated from **Figure 1A** respectively), indicating dysbiosis of the supragingival microbiota of the MAFLD group.

The PCoA and PLS-DA results demonstrated differences in the community compositions between the two groups (Adonis, *P* = 0.0120). The discriminatory taxa between two groups were identified using LEfSe. At the genus level, *Actinomyces* and *Prevotella 2* had the highest LDA scores in the MAFLD group. *Actinomyces* spp. is a normal resident bacteria of the oral cavity, which exerts important roles in biofilm formation (Polak et al., 2019). *Actinomyces* spp. has been associated with the severity of chronic periodontitis (Cao et al., 2018). *Prevotella 2* is a genus of Gram-negative, anaerobic bacteria that exists in the gut and are relevant to multiple disease states, including an increased lifetime risk of cardiovascular disease (Kelly et al., 2016), ankylosing spondylitis (Chen et al., 2019), and increased levels of C-reactive protein (Sun et al., 2019). Considering the consistency between the oral and gut microbiotas (Segata et al., 2012; Arimatsu et al., 2014; Li et al., 2019), the prevalence of *Prevotella 2* in the oral cavity is proposed as a potential marker of systematic diseases including MAFLD. In healthy participants, the genera *Neisseria* and *Bergeyella* had the highest LDA scores. *Neisseria* spp. is among the most abundant taxa in the oral cavity (Dong et al., 2018). A predominance of *Neisseria* spp. in the oral cavity indicates healthy conditions of the oral cavity (Meuric et al., 2017; Yamashita and Takeshita, 2017; Perez-Chaparro et al., 2018). *Bergeyella* spp. is a Gram-negative, aerobic bacteria (Muramatsu et al., 2019). In the present study, *Bergeyella* spp. was more prevalent in the control group, suggesting a negative correlation to MAFLD. Co-occurrence networks were used to predict inter-genera correlations of supragingival plaque between the two groups. As shown in **Figure 3**, there were significant differences in the interaction patterns of the two groups. In the MAFLD group, there were stronger and more complex interactions within the main cluster, but weaker correlations among the genera outside of the main cluster. Reportedly, an increase in interaction strength among taxa not only excludes other taxa, but decreases the stability of the microbial community (Ratzke et al., 2020). Therefore, it could be speculated that the supragingival microbial community of the MAFLD group was more unstable.

Inhibition of hepatic glucose production, increased accumulation of lipids in the liver, and IR are vital to the development of MAFLD (Bessone et al., 2019). It is currently believed that IR is an independent risk factor for the severity of MAFLD (Bessone et al., 2019). As first proposed by Matthews et al. in 1985, HOMA-IR is both practical and highly efficient for the evaluation of IR in both clinical and scientific studies (Matthews et al., 1985; Tang et al., 2015). In the present study, HOMA-IR was beyond the normal range (normal range ≤1) in the MAFLD group and significantly higher than that in the control group (*P* = 0.0013) suggesting that IR is prevalent in patients with MAFLD. It was believed that chronic low-grade inflammation resulting from dysbiosis of the oral microbiota can reportedly aggravate IR (Bui et al., 2019). In this study, Spearman’s correlation analysis revealed that the presence of *Granulicatella* spp., *Veillonella* spp., *Streptococcus* spp., and *Scardovia* spp. was positively correlated with HOMA-IR. It was reported that *Granulicatella* spp. has been positively correlated to periodontitis (Belstrøm et al., 2014), as well as infections outside of the oral cavity, such as infective endocarditis (Sasaki et al., 2020). *Veillonella* is a genus of Gram-negative anaerobic bacteria mainly found in the oral and gastrointestinal tracts. The presence of *Veillonella* spp. in the oral cavity has been correlated to increased production of pro-inflammatory cytokines (Bui et al., 2019; Li et al., 2020) and periodontal infections (Yamashita and Takeshita, 2017). *Streptococcus* spp. and *Scardovia* spp. are resident bacteria of the oral cavity that are closely related to caries formation (Kressirer et al., 2017). Although relatively few studies have investigated the relationship between caries-related bacteria and IR, patients with IR tend to have more decayed teeth (Loyola-Rodriguez et al., 2011).

In a state of chronic low-grade inflammation (Mervish et al., 2019), obesity is a contributor to various metabolic dysfunctions, such as MAFLD and type 2 diabetes (Canfora et al., 2019). As compared to BMI, visceral adiposity, as measured by waist circumference, has been closely linked to the severity of lipid deposition in the liver (Mervish et al., 2019), which is consistent with the results of the present study, which found an increase in waist circumference in MAFLD patients (*P* = 0.0020). In addition, multiple studies have verified the influence of obesity on the microbial profile of the oral cavity (Tam et al., 2018; Chattopadhyay et al., 2019). In this study, genera positively correlated with obesity mainly included *Streptococcus*, *Oslenella*, *Scardovia*, and *Selenomonas*. *Streptococcus* spp. and *Scardovia* spp. were also positively correlated to IR, supporting the positive association between obesity and IR (Mervish et al., 2019). The involvement of *Oslenella* spp. in endodontic infections (Vieira Colombo et al., 2016) and periodontal inflammation (Chen et al., 2020) have been well documented. In the *Veillonellaceae* family, *Selenomonas* is a genus of Gram-negative anaerobic bacteria. Members of *Veillonellaceae* family are considered to act as pro-inflammatory mediators (Tanner, 2015) and putative periodontal pathogens (Rocas and Siqueira, 2005). These results support the presumption that obesity is positively correlated to the abundance of bacteria associated with infectious diseases of the oral cavity (Maciel et al., 2016).

Dyslipidemia is a common clinical manifestation of MAFLD, especially hypertriglyceridemia and low serum HDL-C (Pacifico et al., 2014; Fukuda et al., 2016; Fan et al., 2019), which were also verified in this study (*P* = 0.0083 for TG; *P* = 0.0011 for HDL-C). Reportedly, oral infectious diseases and dyslipidemia could have a two-way relationship without a clear cause-and-effect relationship (Jaramillo et al., 2013). *Actinomyces* spp. has been positively correlated to TG levels as a potential indicator of MAFLD-related metabolic dysfunction. A surprising result was that the presence of *Aggregatibacter* spp. was negatively correlated with TG levels, but positively correlated with HDL-C levels, which might indicate good health, challenging the mainstream concept that the presence of *Aggregatibacter* spp. (especially *A. actinomycetemcomitans*) is related to dyslipidemia and other metabolic diseases (Jaramillo et al., 2013). Sampling sites may explain this discrepancy because *Aggregatibacter* spp. is a anaerobic bacteria with growth behaviors that may change in response to aerobic conditions (supragingival habitats). However, the exact reasons for this paradox remain unclear.

Known as indicators of hepatocellular damage, elevated serum levels of transaminases and transpeptidases are also main clinical manifestations of MAFLD (Sheka et al., 2020). Moreover, a decreased AST/ALT ratio is regarded as biomarker of progressive MAFLD (Sheka et al., 2020). In this study, a decreased AST/ALT ratio (*P* = 0.0142) as well as elevated GGT (*P* = 0.0084) were prevalent in MAFLD patients, suggesting the enrolled MAFLD patients had different degrees of hepatocellular damage. *Capnocytophaga* is a genus of Gram-negative anaerobic bacteria reportedly associated with periodontitis (Cao et al., 2018) and hyperglycemia (Graves et al., 2019). In this study, an abundance of *Capnocytophaga* spp. was negatively correlated to the AST/ALT ratio, suggesting it could be a potential biomarker of MAFLD progression.

Metagenomic predictions based on PICRUSt2 revealed that functional changes between the control and MAFLD groups mainly involved metabolism (**Figure 5B**). Among the KEGG pathways level 3, metabolism of sugars (mainly free sugars, including starch and sucrose, fructose and mannose, and galactose) were more prevalent in subjects with MAFLD, revealing that supragingival plaque in MAFLD patients can easily obtain nutrients, which could explain the increased microbial diversity observed in the supragingival plaque of the MAFLD group (**Table 2**). Pathways related to aerobic respiration (including pyruvate metabolism, and the citrate cycle) were more abundant in the supragingival plaque of the control group, suggesting that the proportion of aerobic bacteria in the supragingival plaque is higher in healthy people. However, a deficiency of predicting functions based on taxa composition is that bacterial functions can change with the health status of the host (Ikeda et al., 2020). Consequently, metatranscriptomics and metabolomics of the microbiota may provide more realistic functional profiles.

As this is a pilot study with matching of confounding factors, some intriguing findings surfaced, but still need to be verified in future studies with larger sample sizes. In addition, with the increasing attention to the functions of the oral microbial community, it is essential to identify changes to the actual functional profiles of the supragingival microbiota in MAFLD by metatranscriptomics and metabolomics.

## Conclusions

In the present study, dysbiosis of supragingival microbiota associated with MAFLD was characterized. Briefly, the results revealed increases in the abundances of bacteria associated with oral infections, decreases in the abundances of potential beneficial aerobic bacteria, and changes in the interactions of the core microflora with the supragingival microbiota in patients with MAFLD. Moreover, as risk factors for the development of MAFLD, IR was positively correlated to the abundances of *Granulicatella* spp., *Veillonella* spp., *Streptococcus* spp., and *Scardovia* spp., while obesity was positively correlated with the abundances of *Streptococcus* spp., *Oslenella* spp., *Scardovia* spp., and *Selenomonas* spp. The increased free sugar metabolic pathways suggested that supragingival bacteria related to the metabolism of free sugars were associated with MAFLD. These findings provide a deeper understanding of the association between the oral microbiome and MAFLD, although further studies are needed to explore potential causal relationships.

## Data Availability Statement

The datasets presented in this study can be found in online repositories. The names of the repository/repositories and accession number(s) can be found below: https://www.ncbi.nlm.nih.gov/, PRJNA645880.

## Ethics Statement

The studies involving human participants were reviewed and approved by the Ethics Committee of Shanghai Ninth People’s Hospital affiliated with Shanghai Jiao Tong University, School of Medicine. The patients/participants provided their written informed consent to participate in this study.

## Author Contributions

FZ and TD performed the study design, data analysis, drafting, revising, final approval, and handled the accountability of all aspects of the work. K-YY and RM performed the bioinformatics analysis and data acquisition. N-JW and F-ZX performed the data analysis and acquisition. RM, Y-LL, DL, Z-MW, and Z-WH performed the study design, clinical sample collection, data analysis, revising, final approval and were also involved in the accountability of all aspects of the work. All authors contributed to the article and approved the submitted version.

## Funding

This work was supported by grants from the Shanghai Top Priority Clinical Medicine Center (no. 2017ZZ01011), the Shanghai Municipal Key Clinical Specialty (no. shslczdzk1601), the Shanghai Clinical Research Center for Oral Diseases (no. 19MC1910600), the National Natural Science Foundation of China (no. 82071104/81570964/81371143), and partly supported by the Shanghai Ninth People’s Hospital affiliated with Shanghai Jiao Tong University, School of Medicine (no. JYJC201806).

## Conflict of Interest

The authors declare that the research was conducted in the absence of any commercial or financial relationships that could be construed as a potential conflict of interest.
