# Supplementary material for: Shifts in the Bacterial Community of Supragingival Plaque Associated With Metabolic-Associated Fatty Liver Disease
Source: Front Cell Infect Microbiol. 2020 Dec 15;10:581888. doi: 10.3389/fcimb.2020.581888 (PMC7770214; doi:10.3389/fcimb.2020.581888)

Supplementary Material

Supplementary Table S1. The purity and concentration of the extracted DNA tested with NanoDrop 2000 UV-vis spectrophotometer (M: MAFLD; H: Health).

| Sample ID | Concentration(ng/μl) | OD260/280 |
| --- | --- | --- |
| M1 | 18.70 | 1.92 |
| M2 | 52.80 | 1.98 |
| M3 | 21.60 | 1.96 |
| M4 | 18.90 | 1.99 |
| M5 | 33.90 | 1.93 |
| M6 | 56.90 | 1.95 |
| M7 | 7.70 | 2.45 |
| M8 | 29.00 | 1.99 |
| M9 | 8.30 | 2.52 |
| M10 | 19.80 | 2.11 |
| M11 | 70.20 | 1.90 |
| M12 | 41.00 | 1.97 |
| M13 | 22.80 | 2.01 |
| M14 | 9.10 | 2.38 |
| M15 | 34.90 | 2.01 |
| M16 | 36.20 | 1.97 |
| M17 | 19.50 | 2.05 |
| M18 | 33.60 | 1.93 |
| M19 | 16.30 | 1.97 |
| M20 | 8.70 | 2.02 |
| M21 | 23.30 | 1.92 |
| M22 | 25.70 | 2.04 |
| M23 | 45.00 | 2.01 |
| M24 | 59.90 | 1.92 |
| H1 | 101.90 | 1.93 |
| H2 | 36.40 | 1.90 |
| H3 | 21.90 | 1.98 |
| H4 | 50.60 | 1.91 |
| H5 | 121.10 | 1.94 |
| H6 | 46.80 | 1.95 |
| H7 | 128.30 | 1.90 |
| H8 | 79.30 | 1.82 |
| H9 | 18.00 | 2.08 |
| H10 | 65.20 | 1.95 |
| H11 | 9.90 | 1.86 |
| H12 | 18.40 | 1.94 |
| H13 | 36.40 | 1.91 |
| H14 | 16.70 | 1.81 |
| H15 | 48.30 | 1.91 |
| H16 | 36.20 | 2.00 |
| H17 | 35.10 | 1.82 |
| H18 | 100.50 | 1.71 |
| H19 | 14.50 | 2.02 |
| H20 | 3.90 | 1.92 |
| H21 | 5.10 | 1.90 |
| H22 | 18.80 | 1.93 |

**Supplementary Figure S1.** The results of 2% agarose gel electrophoresis of the PCR products (A: MAFLD group; B: Health group).


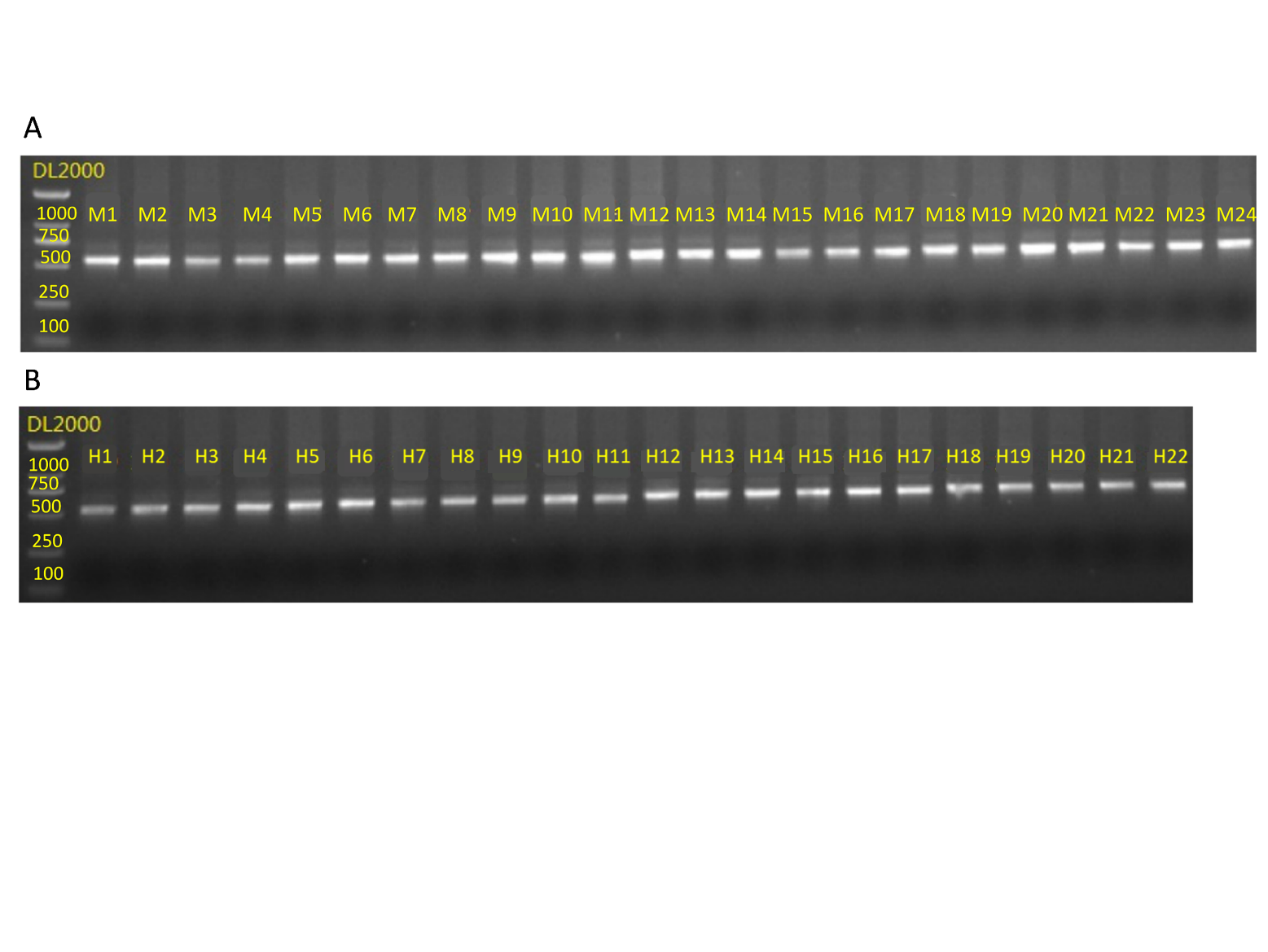


**Supplementary FigureS2.** Rarefaction curves based on OUT level.

**Supplementary Figure S3.** Community heatmap at genus level.

**Supplementary Figure S4** Histograms of the logarithmic linear discriminant analysis (LDA) scores were calculated for the selected phylum (A), class (B), orders(C) and families (D) (logarithmic LDA＞2.0, P＜0.05).


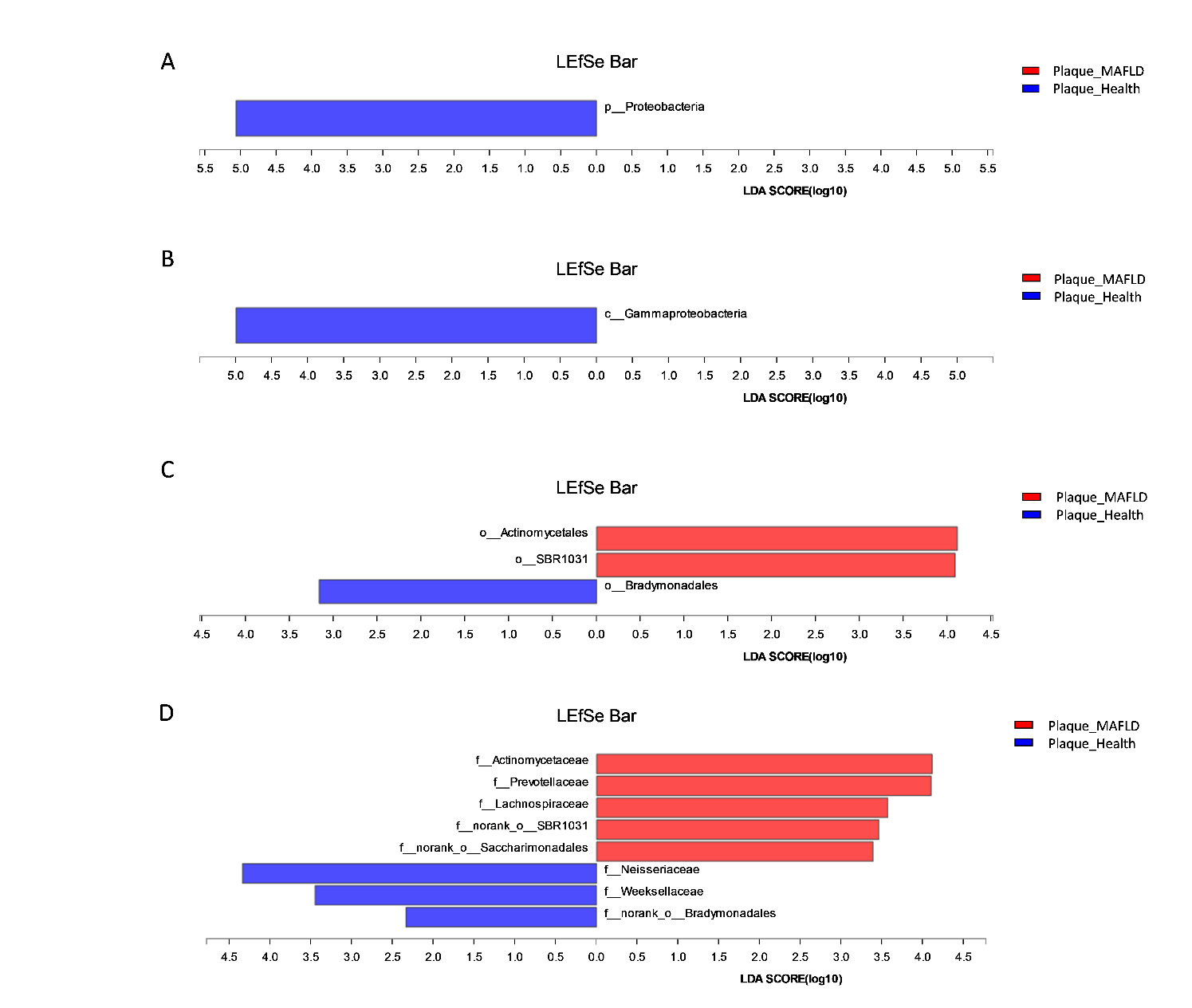

Supplement: Supplementary file 1 [file DataSheet_1.docx]
